# Effect of Padzahr Tablet on Biochemical Indices of Bone Remodeling in Postmenopausal Females with Osteopenia: A Randomized Double-Blind Placebo-Controlled Trial

**DOI:** 10.31661/gmj.v13i.2950

**Published:** 2024-02-08

**Authors:** Shabnam Rafiee, Arash Hossein-nezhad, Zhila Maghbooli, Arman Zargaran, Solaleh Emamgholipour, Afsaneh Ghasemi, Mehrnoosh Ahmadi, Hadi Esmaeeli, Mehrdad Karimi

**Affiliations:** ^1^ Department of Traditional Medicine, School of Persian Medicine, Tehran University of Medical Sciences, Tehran, Iran; ^2^ Section of Endocrinology, Diabetes, Nutrition, and Weight Management, Department of Medicine, Boston University School of Medicine, Boston, MA, USA; ^3^ MS Research Center, Neurosciences Institute of Tehran University of Medical Sciences, Tehran, Iran; ^4^ Department of Traditional Pharmacy, School of Persian Medicine, Tehran University of Medical Sciences, Tehran, Iran; ^5^ Department of Clinical Biochemistry, School of Medicine, Tehran University of Medical Sciences, Tehran, Iran; ^6^ Shahid Akbarabadi Clinical Research Development Unit ( ShACRDU), School of Medicine, Iran University of Medical Sciences, Tehran, Iran; ^7^ Quality Assurance Department, NIAK Pharmaceutical Company, Golestan, Iran

**Keywords:** Padzahr, Women, Osteopenia, Clay, Persian Medicine, Bone

## Abstract

Background: Osteoporosis is a complex disease that poses major global public health challenges. Many individuals with osteoporosis turn to complementary and alternative medicine (CAM) for prevention and management. Due to its mineral contents, Padzahr, a type of clay used in traditional Persian medicine, is believed to have bone-forming properties. This study examined the impact of Padzahr on bone remodeling in postmenopausal women with low bone density.Materials and Methods: In this randomized double-blind and placebo-controlled clinical trial, 48 postmenopausal women with osteopenia were included. The participants were divided into two groups, with 24 participants in each group. One group received Padzahr, and the other group received a placebo. The participants took their assigned treatment for 12 weeks. Blood samples were taken from participants at the study’s beginning and end to compare the two groups’ serum levels of bone remodeling biomarkers. Results: At the outset of the study, the two groups were similar and there were no significant differences in any of the measured variables. Additionally, the levels of bone turnover markers were not significantly different between the two groups at the start of the study (P0.05). After 12 weeks of treatment, the results of the ANCOVA analysis showed no significant changes in the serum levels of bone turnover indices when comparing the Padzahr group to the placebo group (P0.05). Conclusion: A clinical trial of 3 months of Padzahr treatment in postmenopausal women with osteopenia did not show significant changes in serum markers of bone turnover.

## Introduction

Osteoporosis is a condition that weaken bones and makes, them more prone to fractures. This is caused by a reduction in bone density and mass, as well as the deterioration of bone tissue [1]. It is estimated that the prevalence of osteoporosis is over 200 million people worldwide [2]. While about 1 in 5 men over the age of 50 deal with osteoporotic fracture, this rate is 1 in 3 in women [2]. Osteoporosis is a major public health issue, with the World Health Organization (WHO) ranking it as the second most significant health problem after cardiovascular diseases [3].

The pathogenesis of osteoporosis involves a wide variety of constant and variable factors, some of which play crucial roles [4]. Several factors, including heredity, race, aging, gender, and menopause, are inevitable contributors to the onset of osteoporosis [5]. However, other factors, including inadequate vitamin D and calcium intake, a sedentary lifestyle, low body weight, smoking, excessive alcohol consumption, and hormonal disorders, can be modified [6]. Postmenopausal women are at an increased risk of osteoporosis because estrogen, a hormone that helps maintain bone health, declines after menopause [7].

Osteoporosis can be controlled with several therapies, including both pharmacological and non-pharmacological treatments [8]. Lifestyle modifications such as adequate vitamin D and calcium intake, weight-bearing exercises, and reduced smoking and alcohol consumption can be effective in reducing the risk of osteoporosis [8]. In addition, medications including bisphosphonates, estrogen replacement therapy, and raloxifene are effective therapies [9]. Studies have demonstrated that appropriate treatment can decrease the risk of fractures in people with osteoporosis by up to 70% [10]. However, the adherence rate to these treatments is often low, possibly due to the long duration of intervention, financial burden, low availability, and concerns about potential side effects [11]. Although there is no clear evidence, the use of complementary and alternative medicines (CAMs) has become more common in patients with osteoporotic [12].

Clays have long been used as a modality of complementary and alternative medicines and are still used for various purposes, including cosmetics, nutrition, and treatment worldwide [13]. For instance, evidence dating back to about 2500 BC indicated that clays were used for healing wounds and stopping bleeding [13]. Traditional Persian Medicine (TPM) has introduced a type of clay called Padzahr, which is claimed to be effective in treating various conditions, including anemia, uterine bleeding, psychological distress, and osteoporosis, among others. Padzahr is composed of Bezoar, Armenian bole, and Russian clay.

In a search through the literature, we didn’t find any study assessing the effectiveness of Padzahr tablets on bone remodeling. So this study aimed to fill this gap in the literature by investigating the potential impact of Padzahr tablets on osteopenic patients.

## Materials and Methods

Study Design

To investigate the effectiveness of a Padzahr supplement, a 12-week study was conducted at Akbarabadi Hospital affiliated with the Iran University of Medical Sciences from November 2019 to June 2020. This study was a randomized, double-blind, trial, with participants being randomly assigned to either the treatment or control group.

The study was performed based on the guidelines of the Declaration of Helsinki, and the protocol was verified by the ethics committee of the Tehran University of Medical Sciences (IR.TUMS.VCR.REC.1397.422). Additionally, the trial was registered in the Iranian Registry of Clinical Trials (IRCT20180710040406N1) to ensure transparency and accountability.

Before enrollment, all participants were provided with an informed consent form to read and sign, which detailed the purpose and procedures of the study, as well as any potential risks or benefits. The anonymity and confidentiality of all participants were strictly maintained throughout the study.

Participants

The study enrolled postmenopausal women aged 45 to 65 who had been diagnosed with osteopenia, a condition in which bone mineral density (BMD) is lower than normal but not as low as in osteoporosis. Osteopenia is defined by WHO as having a BMD T score between -1 and -2.5 Standard Deviation (SD).

Participants were not eligible to participate in the study if they had any of the following conditions: smoking, alcohol use, a history of bone diseases other than osteopenia, or any of the following critical chronic conditions: cancer, cardiovascular diseases, diabetes, kidney failure, liver disease, systemic inflammatory disease, degenerative joint diseases, and rheumatologic disorders, gastrointestinal diseases, thalassemia, hyperthyroidism, hypogonadism, or Cushing syndrome.

Additionally, those who had taken medications affecting bone metabolism, including bisphosphonates, NSAIDs (non-steroidal anti-inflammatory drugs), diuretics, anticonvulsants, corticosteroids, or HRT (hormone replacement therapy) within the past 6 months, were excluded from the study. Participants with physical or mental health conditions that could interfere with the study, such as motor disabilities, skeletal disorders, or untreated psychiatric illnesses, or those who were unwilling to accept randomization were also excluded. Finally, participants who were taking another intervention, had experienced fractures, did not intend to pursue the study, or had significant side effects during the trial were not included in the trial.

Sample Size Estimation

The sample size for this trial was calculated using G*power software (version 3.1.9), which is a widely used statistical power and sample size calculator [14]. A moderate effect size (d=0.5) and a statistical power of 0.85 at a significant level of 0.05 were used to calculate the required sample size. This resulted in a total of 40 patients (20 per group) needed for the study. To account for potential participant dropout, a 20% loss was assumed, leading to a final sample size of 24 patients in the Padzahr group and 24 patients in the placebo group.

Randomization and Intervention

In this study, patients were randomly assigned to either the groups of the Padzahr or the placebo group equally using a block random sampling method with a 1:1 allocation ratio in blocks of four. The allocation sequence was generated by a computer using a random numbers table to ensure that the allocation was truly random. Randomization was conducted by an independent person who was not involved in the study procedure and was blinded to the group assignment. Both the participants and the investigators were kept unaware of the group assignment to prevent bias in the study.

Participants in the intervention group were instructed to consume two tablets of Padzahr three times a day, with two tablets taken after each meal of breakfast, lunch, and dinner, for a total of 12 weeks. Meanwhile, participants in the other group consumed the same number of placebo tablets during the same period. Both the intervention and placebo tablets were distributed to participants during monthly visits, with each bottle containing 180 tablets, enough for a month’s supply.

This dosage of the drug was determined based on the Persian medicine texts, pre-clinical evaluation based on the experience of researchers, and considering the amount of minerals in the pill to be lower than their toxic level.

To monitor compliance, participants were asked to return the empty bottles to the researchers as an indicator of medication intake. To enhance therapy adherence, the study protocol included a weekly telephone call to participants by one of the investigators. The purpose of these calls was to provide support and encouragement to the participants, as well as to address any concerns they may have had regarding the study or their medication. During these calls, the investigator reminded participants about the importance of taking their medication as prescribed and asked if they had experienced any adverse effects or difficulties using the medication.

Padzahr Composition

The placebo tablets contained lactose and starch and were similar to Padzahr tablets in terms of color, size, and bottle. Both Padzahr and placebo tablets were supplied by Niak Pharmaceutical Company.

Padzahr is a product derived from Traditional Persian Medicine, composed of three primary components: Bezoar (Iron and Magnesium Silicate), Armenian clay (Iron Oxide and Calcium Carbonate), and Russian clay (Aluminium Silicate). The elemental composition of the Padzahr tablet was determined using X-Ray Fluorescence (XRF) analysis, and the amount of each element is presented in Table-1. The placebo tablets used in this study were composed of lactose and starch and were designed to be identical in appearance, size, and packaging to the Padzahr tablets. Both the Padzahr and placebo tablets were supplied by Niak Pharmaceutical Company for use in the study.

Outcome Assessment

A socio-demographic questionnaire was administered to collect general characteristics including age, number of pregnancies, deliveries, and abortions, as well as the age of menarche and menopause. The weight and height of participants were quantified using a digital scale and a stadiometer, respectively. The digital scale was accurate to the nearest 0.1 kg, and the stadiometer was accurate to the nearest 0.5 cm. Participants were barefoot and wearing light clothing during the measurements. The equation of (weight (kg))/(height2 (m)) was applied to estimate the body mass index (BMI).

Dietary intake data were collected using a 24-hour recall tool, and the information was analyzed using a modified version of Nutritionist Software Version IV (First Databank, San Bruno, CA). The level of physical activity was recorded using the International Physical Activity Questionnaire (IPAQ). The DEXA (dual-energy x-ray absorptiometry) was used to quantify Bone Mass Density (BMD) in three areas of the neck, hip, and lumbar.

Ten-milliliter blood samples were taken from patients at the pre-treatment and post-treatment stages to determine the markers of bone turnover. Biochemical bone formation indices included bone alkaline phosphatase (BAP) and procollagen type 1 amino-terminal propeptide (P1NP). Bone resorption markers included C-terminal telopeptide of type I collagen (CTx1). The enzyme-linked immunosorbent assay (ELISA) method was employed to analyze the blood samples to detect bone markers.

Statistic

The Kolmogorov-Smirnov test was implemented to determine the normality of data. To compare the baseline characteristics between the two groups, Independent t-tests or Mann-Whitney U tests were employed depending on whether the data is parametric or non-parametric. To measure the mean differences of parameters between the Padzahr and placebo groups, an ANCOVA model was used with adjustment to baseline score as a covariate. The mean values before and after the intervention were compared with paired t-tests or Wilcoxon tests. The statistical analysis was performed by the SPSS software (version 21; SPSS, Chicago, IL). The statistical significance threshold was set to a P-value of lower than 0.05.

## Results

**Table:** Table1. The Result of Analysis of Padzahr by XRF( X-Ray Fluorescence) Technique

							**L.O.I: 22.5**							
Na2O	MgO	Al2O3	SiO2	P2O5	SO3	Cl	K2O	CaO	TiO2	Cr	Fe2O3	Ni	Sr	Zr
0.449 ^*^	9.67	15.529	44.442	0.179	0.119	0.053	1.244	2.318	0.233	0.059	3.12	0.058	0.007	0.012

^*^Amounts are expressed as a percentage (%) **Na2O:** Sodium Oxide; **MgO:** Magnesium Oxide; **Al2O3:**Aluminium Oxide; **SiO2:** Silicon Oxide; **P2O5:**Phosphorus pentoxide; **SO3:**Sulfur Trioxide; **Cr:** Chromium; **Fe2O3:** Iron Oxide; **Ni:** Nickel; **Sr:** Strontium; **Zr:** Zirconium

**Table:** Table2. Comparison between Padzahr and Placebo Group for General Characteristics, Bone Remodeling and Calcium Homeostasis Markers, Sun Exposure, and Physical Activity of Participants at the Beginning of the Study

	Padzahr group		Placebo group		
	Mean	SD	Mean	SD	P-value
Age (year)	56.60	5.42	54.64	6.47	0.295 ^a^
Height (cm)	158.05	5.07	160.73	4.17	0.068 ^a^
Weight (kg)	74.80	10.29	79.95	9.71	0.103 ^a^
Menarche age (year)	13.15	1.93	13.86	2.93	0.388 ^b^
Menopause Age (year)	46.40	7.21	48.73	4.80	0.463 ^b^
Breastfeeding (month)	69.00	41.22	79.09	48.47	0.570 ^b^
BMI (kg/m2)	25.05	3.50	26.68	3.05	0.114 ^a^
Hip BMD (T-Score)	-0.63	0.62	-0.50	0.58	0.49 ^a^
Lumbar Spine BMD ( T- Score )	-0.95	0.90	-0.82	0.77	0.60 ^a^
Neck of femur BMD ( T- Score)	-1.65	0.49	-1.5	0.68	0.63 ^a^
Total food and drug calcium intake (mg/day)	788.75	193.28	779.66	245.86	0.895 ^a^
Vitamin D Intake IU/day)	3698	84.62	3662	92.11	0.94 ^a^
Sun exposure (min/day)	36.5	35.54	37.95	36.47	0.852 ^b^
Total activity (MET/day)	922.70	775.23	648.40	592.72	0.280 ^b^
BAP (ng/ml)	0.60	0.31	0.47	0.23	0.121 ^a^
P1NP (pg/ml)	455.58	172.63	494.74	174.29	0.485 ^a^
CTx1 (ng/ml)	0.41	0.07	0.42	0.06	0.948 ^a^
OC (ng/ml)	49.87	15.06	48.64	7.61	0.930 ^b^
PTH (pg/ml)	20.35	6.95	27.05	21.91	0.173 ^b^
Serum Calcium (mg/dl)	9.36	0.61	9.53	0.47	0.361 ^b^
Serum Phosphorus (mg/dl)	3.52	0.60	4.03	0.93	0.053 ^b^
VitD (ng/ml)	40.99	12.21	41.68	23.48	0.669 ^b^

**a:** Independent t-test; **b:** Mann-Whitney U test **BAP:** Bone alkaline phosphatase; **P1NP:** Procollagen type I N-terminal propeptide ; **CTX1:** Type I Collagen Cross-Linked C-Telopeptide; **OC:** Osteocalcin; **PTH:** Parathyroid Hormone; Body Mass Index; **BMD:** Bone Mineral Density

**Figure-1:**
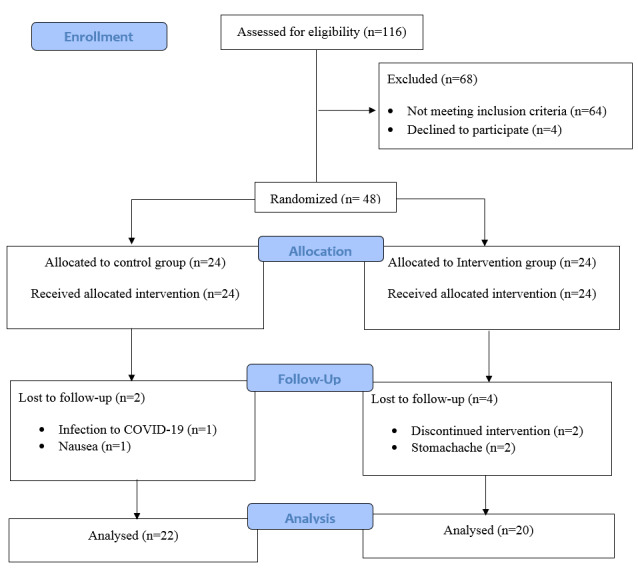
Figure 1. Flow diagram of the study

The study initially screened 116 patients, out of which 68 patients failed to meet the inclusion criteria or declined to participate. The remaining 48 individuals were randomly divided into two groups, with 24 participants in each group receiving either Padzahr or a placebo. Four patients from the intervention group discontinued the trial due to loss of follow-up and stomachache, while two patients from the placebo group withdrew due to coronavirus infection and nausea (Figure-1). The two groups were similar at the start of the study, with no statistically significant differences in any of the variables measured (as shown in Table-2). The average age of participants in the Padzahr group was 56.6±5.42, while this figure in the placebo group was 54.63±6.47.

**Figure-2:**
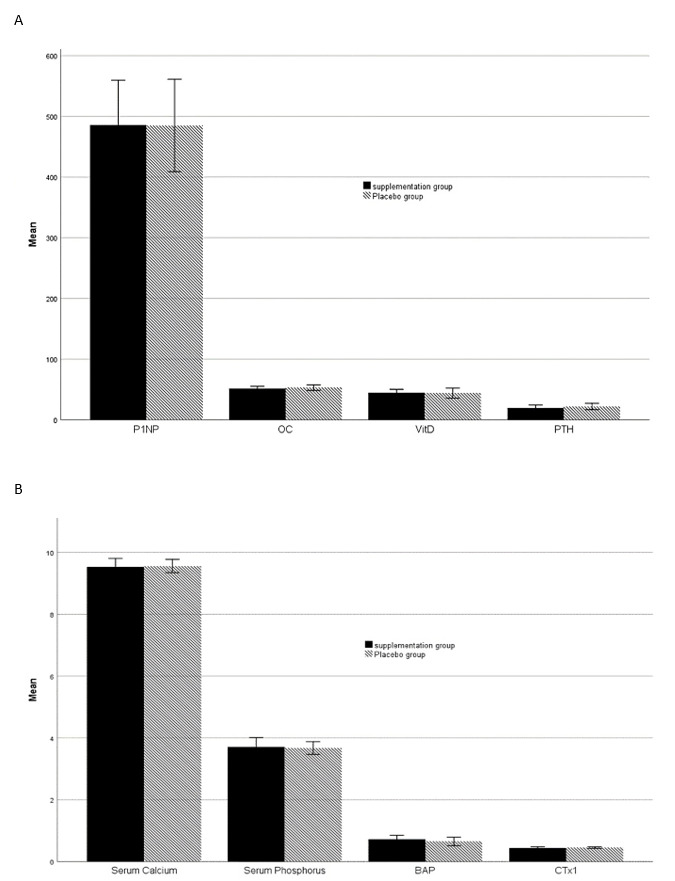
Figure 2. Comparison of bone remodeling and calcium homeostasis marker between Padzahr and placebo group at the end of the trial. Data are shown as Mean ± SD Note: CTX1: Type I Collagen Cross-Linked C-Telopeptide; OC: Osteocalcin; P1NP: Procollagen type I N-terminal propeptide; BAP: Bone alkaline phosphatase; PTH: Parathyroid Hormone; VitD: vitamin D

The bone remodeling markers were not significantly different between the two groups at the start of the study, as presented in Table-2. The ANCOVA analysis indicated that non-significant differences between groups persisted after the intervention, as illustrated in Figure-2. Furthermore, Table-3 provides details on the changes in bone remodeling markers in intra-group comparison at both the baseline and end of the intervention. In the Padzahr group, only the level of BAP increased significantly, while other factors remained stable. Conversely, in the placebo group, the levels of CTx1, Osteocalcin, and BAP were enhanced, and other markers did not change significantly.

## Discussion

**Table:** Table3. Bone Remodeling and Calcium Homeostasis Markers before and after the 12-week in Padzahr or Placebo Group

	Padzahr group		Placebo group		P1	P2
	Mean	SD	Mean	SD		
CTx1 before	0.41	0.07	0.42	0.06	0.177 ^a^	0.008 ^a^
CTx1 after	0.44	0.09	0.46	0.05		
OC before	49.87	15.06	48.64	7.61	0.073 ^b^	0.011 ^a^
OC after	51.46	8.42	53.21	10.19		
P1NP before	455.58	172.63	494.74	174.29	0.132 ^a^	0.464 ^a^
P1NP after	485.54	165.47	485.09	166		
BAP before	0.6	0.31	0.47	0.23	0.018 ^a^	0.003 ^b^
BAP after	0.73	0.29	0.66	0.28		
PTH before	20.35	6.95	27.05	21.91	0.562 ^b^	0.242 ^b^
PTH after	19.67	10.96	24.41	14.47		
Serum Calcium before	9.36	0.61	9.53	0.47	0.379 ^b^	0.844 ^b^
Serum Calcium after	9.53	0.61	9.5	0.48		
Serum Phosphorus before	3.52	0.60	4.03	0.93	0.396 ^a^	0.097 ^b^
Serum Phosphorus after	3.7	0.68	3.7	0.44		
Vitamin D before	40.99	12.21	41.68	23.48	0.215 ^a^	0.66 ^a^
Vitamin D after	44.76	12.31	43.04	17.02		

**P1:** Supplement; **P2:** Placebo **a:** Paired Samples t-test, **b:** Wilcoxon Signed Ranks Test **CTX1:** Type I Collagen Cross-Linked C-Telopeptide; **OC:** Osteocalcin; **P1NP:** Procollagen type I N-terminal propeptide; **BAP:** Bone alkaline phosphatase; **PTH:** Parathyroid Hormone

This trial was conducted for the first time to assess the anti-osteoporotic effects of Padzahr in patients suffering from osteopenia. Based on the findings of the current study, the Padzahr supplement could not improve bone remodeling after three months of intervention.

People tend to consume clay for a variety of reasons includingmedicinal use, detoxification, mineral supplementation, etc [15]. For medicinal purposes, clay is used in conditions including diarrhea, constipation, and alimentary detoxifier and possesses anti-parasite, anti-viral, and antibiotic activities. On the other hand, some of the clays contain different minerals, including calcium, magnesium, iron, manganese, potassium, sulfur, silica, and other trace elements, which are vital for health. Therefore, there are specific proportions of minerals in any clays, which are used in different conditions like anemia and other deficiencies in different cultures [15].

Padzahr is a clay composed of three different components, including Bezoar, Russian, and Armenian clays. This product contains essential minerals for bone remodeling, including iron, calcium, magnesium, aluminum, and silicate [16]. Calcium, an essential mineral in the skeletal structure, accounts for 99% of the calcium in the body and is located in bones [17]. In addition, magnesium can change the apatite crystal structure in the bone, and its deficiency is linked to lower levels of vitamin D and PTH, as well as higher inflammation, which plays a role in osteoporosis [18]. Iron, as a vital mineral, has an essential role in vitamin D metabolism via the cytochromes P450 and hydroxylation of lysyl and prolyl, and could contribute to bone turnover [19]. Moreover, numerous studies have shown that silicon can have beneficial effects on bone formation, leading to increased bone mineral density [20][21]. Although the results of this trial did not find positive effects of these minerals as clay in the treatment of osteoporosis, this may be due to the form of intervention, duration, or amount of mineral in clay. In contrast to our study, the results of an in vitro and in vivo study revealed that nano-montmorillonite, which is the main component of bentonite, a therapeutic clay, could suppress osteoclastogenesis and incite osteoblastogenesis, and consequently improve bone formation [22]. Clays have been used for medicinal purposes for centuries in different cultures, although there is still no evidence-based mechanism [23]. There is a wide variety of clays in different regions, and Galen (130-210), a Greek physician, introduced a type of clay known as Lemons, which was applied for the treatment of diarrhea, ulcers, and animal bites [24]. This clay was also mentioned in the works of other ancient physicians, such as Avicenna (980-1037) and Ibn al-Baitar (1197-1248). Armenian bole is one of the most famous edible and therapeutic clays, which is one of the components of Padzahr [25]. It belongs to the Armenia region and has been used in disorders including diarrhea, dysentery, hemorrhage, and so forth [13].

Moreover, clays could possess anti-bacterial and anti-inflammatory properties. Efimenko et al. conducted an experimental study in rats and concluded that Tambukan clay, obtained from a lake in Russia, possesses anti-inflammatory effects [26]. In addition, in a trial by Miller et al., findings demonstrated that treatment with Sierrasil, a natural mineral product extracted from the Sierra Mountains in the USA, alone or along with a cat’s claw, can improve joint function and decrease pain [27]. In another intervention, this product was effective in improving the quality of life, physical activity, and physical performance of patients with osteoarthritis [28]. owed that certain clays can inhibit the growth of antibiotic-resistant bacteria, including methicillin-resistant Staphylococcus aureus [29]. Furthermore, Afro-Americans have used clay to improve wound healing in patients infected with bacillus Mycobacterium ulcerans [30].

On the other hand, the consumption of clays is not completely safe and could be accompanied by some side effects. For instance, the intake of some clays can lead to gastrointestinal disorders, including constipation, or interfere with the absorption of some nutrients [13]. In addition, some of these edible clays contain heavy metals like Pb, Hg, Cr, Sb, and As, which are toxic and, consequently, can have detrimental effects on health [31].

The following limitations were identified in the current study: 1) the low number of sample size, 2) the process of bone remodeling takes a long time, particularly with age increase. Therefore, a lack of efficacy may be due to short-term intervention. 3) this supplement should be used with a lot of water to have better efficacy, while there is no information about it.

## Conclusion

The study’s findings indicated that over three months, the use of the Padzahr tablet did not improve bone formation among postmenopausal women with osteopenia. However, due to study limitations, further research is needed to evaluate the efficacy of this therapeutic clay in a variety of clinical settings with different age groups, longer duration, and other medical conditions.

## Acknowledgment

The authors would like to thank the Shahid Akbarabadi Clinical Research Development Unit (ShACRDU), Iran University of Medical Sciences (IUMS), Tehran, Iran, for their assistance, support, and kindness throughout the study (IR.TUMS.VCR.REC.1397.422). This study was supported by the Tehran University of Medical Sciences.

## Conflict of Interest

The authors declare no conflicts of interest to any party.
